# Evaluating the cost-effectiveness of [^18^F]FDG-PET/CT for investigation of persistent or recurrent neutropenic fever in high-risk haematology patients

**DOI:** 10.1186/s40644-023-00647-7

**Published:** 2023-12-15

**Authors:** Michelle Tew, Abby P. Douglas, Jeff Szer, Ashish Bajel, Simon J. Harrison, Shio Yen Tio, Leon J. Worth, Rodney J. Hicks, David Ritchie, Monica A. Slavin, Karin A. Thursky, Kim Dalziel

**Affiliations:** 1 Health Economics Unit, Centre for Health Policy, Melbourne School of Population and Global Health, University of Melbourne, Melbourne, Australia; 2 National Centre for Infections in Cancer, Peter MacCallum Cancer Centre, Melbourne, Australia; 3 Department of Infectious Diseases, Peter MacCallum Cancer Centre, Melbourne, Australia; 4 Department of Health Services Research and Implementation Science, Peter MacCallum Cancer Centre, Melbourne, Australia; 5 Department of Oncology, University of Melbourne, Melbourne, Australia; 6 Department of Medicine, University of Melbourne, Melbourne, Australia; 7 National Centre for Antimicrobial Stewardship, The Peter Doherty Institute for Infection and Immunity, Melbourne, VIC Australia; 8 Victorian Infectious Diseases Service, Royal Melbourne Hospital, Melbourne, VIC Australia; 9 Department of Clinical Haematology, Peter MacCallum Cancer Centre and the Royal Melbourne Hospital, Melbourne, VIC Australia

**Keywords:** Cost-effectiveness, Costing, Diagnostic imaging, Febrile neutropenia, Antimicrobial

## Abstract

**Background:**

A recent randomised trial demonstrated [^18^F]fluorodeoxyglucose positron-emission tomography in combination with low-dose CT (FDG-PET/CT), compared to standard of care computed tomography (CT) imaging, positively impacted antimicrobial management and outcomes of acute leukaemia and haematopoietic stem cell transplant recipients with persistent and recurrent neutropenic fever. We conducted an economic evaluation from a healthcare perspective alongside the clinical trial.

**Methods:**

Unit costs in Australian dollars were applied to all resources used (antimicrobials, diagnostic tests, ICU and hospital bed days). Effectiveness was measured as number of patients with antimicrobial rationalisation, 6-month mortality and quality-adjusted life years (QALYs) derived from patient-reported trial-based health-related quality-of-life. Generalised linear models were used to analyse costs and outcomes. Incremental cost-effectiveness ratios (ICERs) for all outcomes and net monetary benefit (NMB) for QALYs were calculated. We performed bootstrapping with 1000 replications using the recycled predictions method.

**Results:**

The adjusted healthcare costs were lower for FDG-PET/CT (mean $49,563; 95%CI 36,867, 65,133) compared to CT (mean $57,574; 95% CI 44,837, 73,347). The difference in QALYs between the two groups was small (0.001; 95% CI -0.001, 0.004). When simulated 1000 times, FDG-PET/CT was the dominant strategy as it was cheaper with better outcomes than the standard CT group in 74% of simulations. The estimated NMBs at willingness-to-pay thresholds of $50,000 and $100,000 per QALY were positive, thus FDG-PET/CT remained cost-effective at these thresholds.

**Conclusions:**

FDG-PET/CT is cost effective when compared to CT for investigation of persistent/recurrent neutropenic fever in high-risk patients, providing further support for incorporation of FDG-PET/CT into clinical guidelines and funding.

**Trial registration:**

This trial is registered with ClinicalTrials.gov, NCT03429387.

**Supplementary Information:**

The online version contains supplementary material available at 10.1186/s40644-023-00647-7.

## Introduction

A recent multicentre randomised trial (PIPPIN study) demonstrated that, compared with standard of care computed tomography (CT) imaging, [^18^F]fluorodeoxyglucose positron-emission tomography in combination with low dose computed tomography (FDG-PET/CT) was associated with a higher rate of rationalisation of broad-spectrum antimicrobial agents to narrower spectrum or no agents in acute leukaemia and/or allogeneic haematopoietic stem cell transplant (HSCT) recipients with persistent and recurrent neutropenic fever [1]. In a population with traditionally high rates of exposure to antimicrobials with associated serious consequences such as multi-drug resistant organism colonisation and adverse effects on the gut microbiome, antimicrobial de-escalation is a high priority. Further, a reduced length of stay post scan in the FDG-PET/CT arm was observed, which may indicate another clinical benefit of this diagnostic approach and an avenue for reducing costs of care and alleviate pressures on hospital services which can be reallocated to meet other medical demands.

Despite these demonstrated benefits, FDG-PET/CT is often primarily used as a diagnostic tool for cancer evaluation [2–4]. However, studies have also demonstrated the potential of this imaging modality to be useful in diagnosing and managing a range of opportunistic infections [5–7]. Demonstrated cost-effectiveness would facilitate advancement of funding models to support routine use of FDG-PET/CT in high-risk patients. In this study we present the results of an economic evaluation of the PIPPIN study, assessing whether FDG-PET/CT compared with CT is cost-effective as a diagnostic tool in the management of persistent or recurrent neutropenic fever.

## Methods

### Study design, participants and outcomes

The PIPPIN trial was a multicentre, open-label, phase 3, randomised controlled trial at two tertiary referral centres with an integrated haematology service based in Victoria, Australia. The two centres perform allogeneic HSCT and intensive chemotherapy for acute leukaemia (Royal Melbourne Hospital [RMH]), and autologous HSCT (Peter MacCallum Cancer Centre [PMCC]). Trial participants were aged ≥ 18 years and about to undergo induction, consolidation, or re-induction chemotherapy for acute leukaemia with expected duration of profound neutropenia (≤ 0·5 cells/µl) of at least 10 days, or undergoing conditioning for an autologous or allogeneic HSCT. Exclusion criteria were pregnancy, allergy to iodinated contrast, or an estimated glomerular filtration rate less than 30 mL/min. The primary outcome of the trial was antimicrobial rationalisation, which was a composite endpoint of either commencement of an antimicrobial (antibacterial, antiviral, or antifungal) with targeted treatment intent (start antimicrobial), cessation of all agents in an antimicrobial class (stop antimicrobials) or change in antimicrobial spectrum (subclassified as broadened or narrowed spectrum [8] within 96 h of the performance of a study-specific scan. The full trial protocol and findings have been published [1]. The trial and cost-effectiveness analysis were approved by the Human Research Ethics Committee of Melbourne Health (HREC/17/MH/106) and registered with ClinicalTrials.gov, (NCT03429387).

The economic evaluation was prospectively designed and was performed from a healthcare system perspective alongside the clinical trial. The cost-effectiveness was compared between the FDG-PET/CT group and the CT group over 6 months following the randomised scan. All patients in the per-protocol population were included in the cost-effectiveness analysis as some patients in the intention-to-treat population did not receive the allocated scan, which would lead to a mismatch between resources and outcomes achieved.

### Estimation of costs

Standardised case report forms were used to collect resource utilisation data during the trial period. Specific costs evaluated included types and doses of antimicrobials administered, subsequent investigations conducted, such as blood cultures, other microbiology pathology tests, diagnostic biopsies, bronchoscopies, echocardiograms, and radiologic procedures, and costs related to the period of neutropenic fever up to time of hospital discharge (ICU admissions and hospitalisation). Medication costs were calculated based on unit costs provided by PMCC Pharmacy Department. All other costs were calculated based on unit costs retrieved from public sources for hospitalisation costs for neutropenic fever episodes [9] and Australia’s Medicare Benefits Schedule which provides a list of medical services subsidised by the Australian Government [10]. A full list of the unit cost prices and sources can be found in Appendix 2. Where necessary, costs were adjusted to 2020 prices using the Consumer Price Index from the Australian Bureau of Statistics. Longer term costs (post-discharge) were not considered in this study because in the setting of neutropenic fever, costs in general are likely to be highly concentrated around the index admission and could be confounded by various other factors such as subsequent chemotherapy cycles. All costs were reported in 2020 Australian dollars (AUD$).

### Effectiveness outcomes

The effectiveness outcomes selected for the economic evaluation were number of patients with antimicrobial rationalisation, mortality at 6 months and quality-adjusted life years (QALYs) derived from patient-reported health-related quality-of-life collected during the trial. In both cohorts, EORTC QLQ-C30 questionnaire was used to capture quality-of-life at the day of the scan and at discharge [1]. Responses from the questionnaires were scored as utility values using Australia-specific published valuation sets [11]. Questionnaires were completed by patients who were well enough to do so, and was only introduced at approximately the midpoint of overall patient recruitment, leaving a substantial number of questionnaires incomplete due to either clinical instability or failure to capture. Completed quality-of-life questionnaires were available from 51 patients across both cohorts. As completion rate was low, responses from both cohorts were pooled and used to describe health states related to the hospitalisation and post-discharge to avoid the potential of selection bias. To obtain QALYs for the inpatient stay, pooled utility score reported at the time of scan (0.411, SD 0.212) was multiplied by each patient’s length of hospitalisation. For QALYs post-discharge, pooled utility score reported at discharge (0.585, SD 0.222) was multiplied by number of days between discharge to end of follow-up (6 months) or censored, whichever came first.

### Statistical analyses

Categorical variables were summarised by frequency and percentage and analysed using a χ² test. Continuous variables were summarised with means (SD) or medians (IQR). To compare the cost differences in resources used between the two cohorts, nonparametric bootstrap simulations were performed with 1000 replications to estimate uncertainty levels.

Generalised linear models (GLM) were used to analyse total costs and outcomes. The appropriate family of distribution and link for the models were determined using a combination of statistical tests including the modified Park test, Pearson correlation, Pregibon and modified Hosmer and Lemeshow tests [12]. Age, sex, adjusted Charlson Comorbidity Index, primary disease, prior HSCT and reason for admission (chemotherapy or transplant) were included in the models to control for possible baseline imbalances. The outcomes, costs and differences between the two cohorts were estimated and presented.

Incremental cost-effectiveness ratios (ICERs) for each of the outcomes were calculated as the mean cost difference between the two cohorts divided by the difference in outcomes between cohorts. For the outcomes presented in the study, the ICER can be interpreted as the cost per patient with antimicrobial rationalisation, cost per death averted and cost per QALY gained. To account for sampling, we performed bootstrapping with 1000 replications using the recycled predictions method [12]. The results of the bootstraps were presented in cost-effectiveness planes. Cost-effectiveness planes show differences in costs on the vertical axis and differences in effect on the horizontal axis. The net monetary benefit (NMB) was also calculated for the QALY outcome using the net benefit framework [13]. Positive NMB values indicate that the intervention would be cost-effective, i.e., the benefits of the intervention outweigh its cost. The probability of the intervention being cost-effective will be tested at a different willingness-to-pay (WTP) thresholds. To explore parameter uncertainty, several sensitivity analyses were conducted, in which costs of FDG-PET/CT and hospitalisation and utility values were varied. Post-hoc subgroup analysis by reason of admission (chemotherapy or transplant) was conducted to explore if there were specific populations that would benefit from a more targeted use of the intervention.

The economic evaluation follows the International Society for Pharmacoeconomics and Outcomes Research (ISPOR) guideline for trial-based cost-effectiveness analysis [14] and reporting follows the ISPOR consolidated health economic evaluation reporting standards guideline (Appendix 1) [15].

## Results

### Clinical trial

Between 8 Jan 2018 and 23 July 2020, 147 high-risk haematology patients with persistent or recurrent neutropenic fever were randomised into the PIPPIN trial, with 134 (91%) completing the study per protocol; 65 in the FDG-PET/CT (intervention) group and 69 in the CT (control) group. The baseline characteristics are presented in Table 1. There was a greater proportion of patients with acute lymphoblastic leukaemia and myelodysplastic syndrome in the CT group than in FDG-PET/CT group.

**Table 1:** Baseline characteristics of patients and results of clinical trial

	FDG-PET/CT group (*n* = 65)		Standard CT group (*n* = 69)		
Characteristic	*n*	%	*n*	%	*p*-value
Age median (range), years	55	(19–73)	55	(18–77)	0.70
Female	19	27.5	27	41.5	0.09
Primary underlying disease					0.01
Acute myeloid leukaemia	38	58.5	33	47.8	
Acute lymphoblastic leukaemia	3	4.6	13	18.8	
Myelodysplastic syndrome	3	4.6	10	14.5	
Other	21	32.3	13	18.8	
Reason for admission					0.23
Chemotherapy	34	52.3	29	42.0	
Transplant	31	47.7	40	58.0	
Age-adjusted Charlson Comorbidity Index median (IQR)	3	(2–4)	3	(2–4)	0.89
Prior HSCT	5	7.7	4	5.8	0.66
**Outcomes**					
Antimicrobial rationalisation ^a^	53	81.5	45	65.2	0.03
Length of hospitalisation post scan mean (SD)	15.4	15.3	17.3	16.4	0.50
Intensive care admission post scan	6	9.2	9	13.0	0.48
6-month mortality	8	12.5	10	14.7	0.71

Data are *n* (%) unless otherwise indicated

^a^ Primary outcome of clinical trial

Antimicrobial rationalisation occurred in 53 (81.5%) of 65 participants in the FDG-PET/CT group and 45 (65.2%) of 69 participants in the CT group (difference 16.3%, *p*-value = 0.03), with the predominant difference between groups being a greater narrowing spectrum of therapy in the FDG-PET/CT arm (28 (43.1%) vs. 17 (24.6%), *p*-value = 0.02, odds ratio = 2.31). The median length of hospitalisation as reported in the clinical study was shorter in the FDG-PET/CT group (9 days) compared to 13 days in the standard CT group (*p*-value = 0.016). The mean length of hospitalisation post randomised scan was observed to be shorter in the FDG-PET/CT group compared to the standard CT group (15.4 days [SD 15.3] vs. 17.3 days [SD 16.4], *p*-value = 0.502). FDG-PET/CT and CT randomised scans were reported as clearly negative in 22/65 (33.8%) and 15/69 (21.7%), respectively (*p* = 0.12). As per the adjudication committee’s assessment, the randomised scan led to a new diagnosis or identification of a new site of infection in 25/65 (40%) and 21 /69 (30%) in FDG-PET/CT and CT arms respectively (OR 1.54; 95% CI 0.75, 3.18, *p* = 0.24).

### Cost of resources used

The cost of resources and hospitalisation related to the period of neutropenic fever up to time of hospital discharge are presented in Table 2. On average, patients in the FDG-PET/CT group had a smaller number of diagnostic imaging, invasive diagnostic and microbiology pathology tests (Appendix 3). ICU admission and hospitalisation costs were the main contributors to the total cost, costing $49,354 [SD 58,054] and $52,689 [SD 51,167] in the FDG-PET/CT and CT groups, respectively. Although not statistically significant, ICU admission costs were observed to be substantially higher in the FDG-PET/CT group compared to the CT group. This was largely influenced by one patient in the FDG-PET/CT group who had a prolonged ICU stay (60 days). The impact of this on the cost-effectiveness analysis was tested in sensitivity analysis (Appendix 6).

**Table 2:** Comparison of costs (2020 AUD$) of resources used ^a^

	FDG-PET/CT group (*n* = 65)				Standard CT group (*n* = 69)				Differences ^b^		
	Mean	SD	Median	IQR	Mean	SD	Median	IQR	Mean	95% CI	
**Antimicrobials** ^**c**^	673	906	438	308	504	285	418	225	168	-55	392
**Diagnostic imaging** ^**d**^	1,008	91	953	94	832	852	811	1,152	176	-29	380
**Invasive diagnostics**	70	223	0	0	143	437	0	0	-73	-184	38
**Pathology tests**	233	285	164	303	335	382	217	386	-102	-213	8
**CVC reinsertion**	47	144	0	0	56	222	0	0	-9	-70	52
**ICU**	9,847	43,378	0	0	4,894	20,490	0	0	4,953	-6,632	16,539
**Hospitalisation**	39,507	34,759	29,314	26,383	47,795	45,800	35,177	23,452	-8,288	-21,737	5,160
**Total cost**	51,385	59,007	30,943	26,454	54,560	52,040	40,192	31,052	-3,175	-22,310	15,960

^a^ Not adjusted for baseline differences

^b^ 95% CI generated from 1000 replications using non-parametric bootstrap simulations

^c^ Related to treatment for febrile neutropenia episode

^d^ Includes cost of intervention FDG-PET/CT scan

### Economic evaluation

The results of the cost-effectiveness analyses are presented in Table 3. The adjusted health care costs were lower in the FDG-PET/CT group (mean $49,563; 95% CI 36,867, 65,133) compared to the standard CT group (mean $57,574; 95% CI 44,837, 73,347); the difference was not statistically significant (-$8,011; 95%CI -27,998, 13,986). The magnitude of differences in QALYs between the two groups was small (0.001; 95% CI -0.001, 0.004) and was not statistically significant.

**Table 3:** Results of cost and effectiveness outcomes and cost-effectiveness analyses

	FDG-PET/CT group			Standard CT group			Difference / ICER / NMB		
	Mean	95% CI		Mean	95% CI		Mean	95% CI	
Total cost	49,563	36,867	65,133	57,574	44,837	73,347	-8,011	-27,998	13,986
QALY	0.285	0.283	0.287	0.284	0.282	0.286	0.001	-0.001	0.004
ICER: Cost per QALY gained							FDG-PET/CT dominates ^a^	-23,165,055	30,415,796
NMB at WTP = $50,000							8,082	-13,459	29,624
NMB at WTP = $100,000							8,154	-13,516	29,823
Antimicrobial rationalisation	81.8%	71.9%	91.2%	65.1%	51.7%	76.2%	16.7%	-0.3%	33.0%
ICER: Cost per patient change in antimicrobial therapy							FDG-PET/CT dominates ^a^	-446,892	161,378
Mortality at 6 months	16.3%	6.3%	31.0%	18.6%	7.9%	34.5%	-2.3%	-17.9%	13.1%
ICER: Cost per death averted							FDG-PET/CT dominates ^a^	-1,976,110	2,207,504

^a^ FDG-PET/CT dominates indicates that FDG-PET/CT is less costly and more effective

**Fig. 1:**
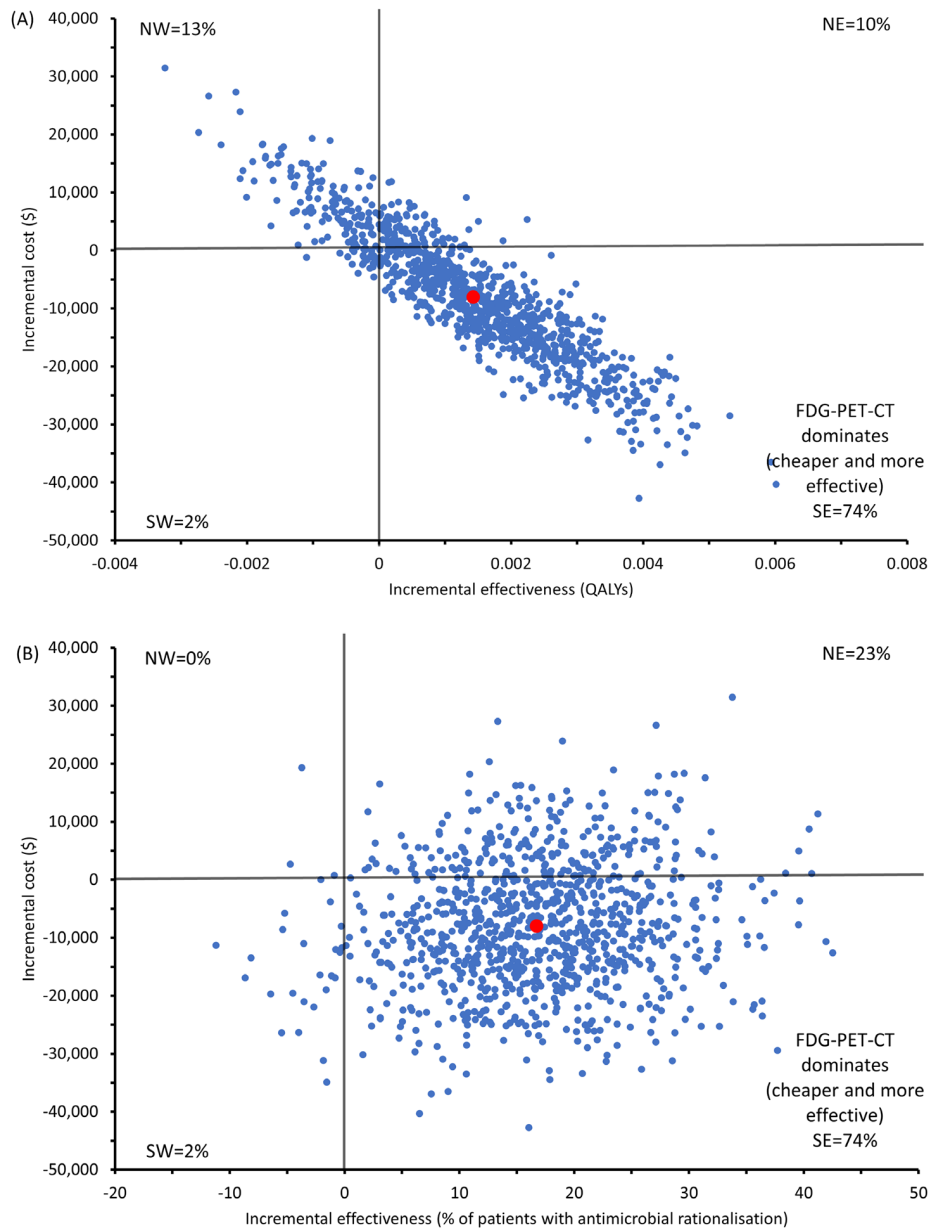
Cost-effectiveness analysis plane for **(A)** QALY and **(B)** antimicrobial rationalisation outcomes. The red dot represents the ICER point estimate which sits in the south-east quadrant, indicating that the FDG-PET/CT intervention is cheaper and more effective compared to standard CT. NW = north-west quadrant; intervention more costly, less effective. NE = north-east quadrant; intervention more costly, more effective. SE = south-east quadrant; intervention less costly, more effective. SW = south-west quadrant; intervention less costly, less effective

Across all three outcomes, the intervention group (FDG-PET/CT) was dominant (Table 3), indicating that it was cheaper and had better outcomes than the CT group. The uncertainty surrounding the expected incremental costs and outcomes, QALY and antimicrobial rationalisation, are presented in Fig. 1A and B, respectively. They show that that for 74% of simulations, the intervention group was dominant compared to standard-of-care group. The cost-effectiveness planes for the 6-month mortality outcome is presented in Appendix 4. The estimated NMBs at WTP thresholds of $50,000 and $100,000 per QALY were positive (Table 3), indicating that FDG-PET/CT remained cost-effective at these thresholds.

The results of the subgroup analysis for both transplant and acute leukaemia chemotherapy patient groups indicate that there was little difference in cost and outcomes between the two groups and FDG-PET/CT remained the dominant strategy (Appendix 5). In sensitivity analysis, despite varying different key components such as increasing the cost of the intervention, hospitalisation and decreasing utility values, the FDG-PET/CT intervention remained the dominant strategy (Appendix 6). Excluding the patient with prolonged ICU stay from the analysis increased the probability of the intervention being dominant from 74 to 94%.

## Discussion

This is the first formal health economic analysis of FDG-PET/CT for management of persistent or recurrent neutropenic fever in high-risk haematology patients. This study demonstrates that FDG-PET/CT is cost-effective as a diagnostic tool and improves outcomes for patients with persistent/recurrent neutropenic fever through improved antimicrobial rationalisation.

Consistent with the original PIPPIN primary and secondary outcome analysis [1], a major factor that contributed to the cost-effectiveness of FDG-PET/CT was the shorter length of stay in this arm. This important finding is likely explained by greater confidence in the findings on FDG-PET/CT, in both ruling in and out infection, and hence guiding de-escalation of therapy, reducing the number of further investigations and reassuring clinicians that a patient is safe for discharge. Correspondingly, other contributory factors to reduced costs in the FDG-PET/CT arm included the trend of lower costs from invasive diagnostics (principally bronchoscopy) and microbiology pathology tests in the FDG-PET/CT arm, which are likely a result of relatively higher rates of possible invasive fungal disease in the CT arm and an overall higher likelihood of needing a bronchoscopy based on non-specific pulmonary parenchymal findings seen on CT. FDG-PET/CT has been shown to be particularly useful in reliably identifying pulmonary invasive fungal disease (IFD) and its dissemination [16] and hence FDG-PET/CT in potential IFD is likely a particularly cost-saving area, which could be explored further in a dedicated study.

Other costing elements were marginally higher in the FDG-PET/CT arm. Diagnostic imaging costs were higher than the CT arm, however this mean difference of $176 is mostly accounted for by the cost-differential between the two interventions themselves (approximately $73 comparing FDG-PET/CT to CT chest, abdomen, pelvis), and therefore to be expected. Additional diagnostic imaging costs in the FDG-PET/CT arm above the intervention itself were small. ICU costs were greater in the FDG-PET/CT arm, however once a significant single outlier was removed (with an ICU length of stay of 60 days), then costs were comparable.

Our results show that FDG-PET/CT remains a cost-effective approach in both transplant and acute leukaemia chemotherapy subgroups. Whilst these groups are both considered high risk of persistent/recurrent neutropenic fever and at risk of certain infections resulting from prolonged neutropenia such as IFD, they can have different clinical characteristics and morbidities/complications (for example, graft versus host disease being particular to the transplant group). It appears that both groups not only derive clinical benefit from the FDG-PET/CT, as evidenced in the PIPPIN study exploratory subgroup analysis [1], but also that cost-effectiveness is likely. This provides necessary support for clinical guidelines, as economic analysis is a key element of feasibility assessment.

Demonstration of cost-effectiveness of FDG-PET/CT is essential for policy makers and funders. The prior notion that FDG-PET/CT is far more costly and will not change management has been disproved in the PIPPIN study, where advantageous change in management (reduced broad spectrum antimicrobial therapy and de-escalation to oral therapy) was demonstrated. This cost-effectiveness analysis confirms that the outlay cost of FDG-PET/CT is covered by the reduced length of stay and other reduced costs observed in those with clearer management plans and de-escalation of treatment.

There are several limitations to this study. The reported wide confidence intervals were indicative of the small sample size, which was expected when we bootstrap from small samples. While we acknowledge that a larger sample size would have been beneficial, this was beyond the scope and pre-determined accrual of the primary trial. Further, this was a study conducted in two centres in Victoria, Australia and findings may not be generalisable to other centres in which the costs of investigations, drugs and hospitalisation may vary. However, we have presented disaggregated costs and outcomes which should facilitate considerations of generalisation. Another limitation is that the current cost-effectiveness analysis conclusions were based on the results over a short time horizon (follow up period of 6 months). In the setting of neutropenic fever, costs in general are likely to be highly concentrated around the index admission, which we have focused on in this analysis. Conversely, the positive outcomes of reduced broad-spectrum antimicrobial use in neutropenic fever are likely to be great and difficult to capture in a short timeframe and in a clinical trial, with reduced antimicrobial resistance, antibiotic-associated infections such as *C.difficile* and adverse effects on the gut microbiome benefiting not only the patient but society more broadly. Increasing access to acute hospital beds for other patients by reducing ICU and overall hospital stays may provide a significant benefit in the context of ongoing stresses on resources such as those related to the COVID-19 pandemic.

## Conclusions

FDG-PET/CT is cost effective when compared to standard of care CT for investigation of persistent/recurrent neutropenic fever in high-risk haematology and HSCT patients. This analysis provides further support for incorporation of FDG-PET/CT into clinical guidelines and for funding models to support this investigation for this indication.

### Electronic supplementary material

Below is the link to the electronic supplementary material.

**Supplementary Material 1**: Appendix 1 – 6

## Data Availability

The datasets generated during and/or analysed during the current study are available from the corresponding author on reasonable request.

## List of Abbreviations

AUD: Australian dollars
CT: Computed tomography
EORTC QLQ-C30: European Organisation for Research and Treatment of Cancer Quality of Life of Cancer Patients
FDG-PET/CT: [^18^F]fluorodeoxyglucose positron-emission tomography in combination with low-dose CT
GLM: Generalised linear model
HSCT: Haematopoietic stem cell transplant
ICER: Incremental cost-effectiveness ratio
ICU: Intensive care unit
IQR: Interquartile range
ISPOR: International Society for Pharmacoeconomics and Outcomes Research
NMB: Net monetary benefit
PMCC: Peter MacCallum Cancer Centre
QALY: Quality adjusted life year
RMH: Royal Melbourne Hospital
SD: Standard deviation
WTP: Willingness-to-pay
