# Supplementary material for: Evaluating the cost-effectiveness of [18F]FDG-PET/CT for investigation of persistent or recurrent neutropenic fever in high-risk haematology patients
Source: Cancer Imaging. 2023 Dec 15;23:119. doi: 10.1186/s40644-023-00647-7 (PMC10724891; doi:10.1186/s40644-023-00647-7)

**Supplementary Materials**

**Appendix 1: CHEERS Checklist**

| **Topic** | **No.** | **Item** | **Location where item is reported** |
| --- | --- | --- | --- |
| **Title** |  |  |  |
|  | 1 | Identify the study as an economic evaluation and specify the interventions being compared. | Title, Page 1 |
| **Abstract** |  |  |  |
|  | 2 | Provide a structured summary that highlights context, key methods, results, and alternative analyses. | Abstract, Page 2 |
| **Introduction** |  |  |  |
| **Background and objectives** | 3 | Give the context for the study, the study question, and its practical relevance for decision making in policy or practice. | Introduction, Paragraph 2 |
| **Methods** |  |  |  |
| **Health economic analysis plan** | 4 | Indicate whether a health economic analysis plan was developed and where available. | Yes, written in the clinical trial protocol. |
| **Study population** | 5 | Describe characteristics of the study population (such as age range, demographics, socioeconomic, or clinical characteristics). | Methods, Paragraph 1 |
| **Setting and location** | 6 | Provide relevant contextual information that may influence findings. | Methods, Paragraph 1 |
| **Comparators** | 7 | Describe the interventions or strategies being compared and why chosen. | Methods, Paragraph 2 |
| **Perspective** | 8 | State the perspective(s) adopted by the study and why chosen. | Methods, Paragraph 2 |
| **Time horizon** | 9 | State the time horizon for the study and why appropriate. | Methods, Paragraph 2 |
| **Discount rate** | 10 | Report the discount rate(s) and reason chosen. | Not reported as not required due to short time horizon |
| **Selection of outcomes** | 11 | Describe what outcomes were used as the measure(s) of benefit(s) and harm(s). | Methods, Paragraph 4 |
| **Measurement of outcomes** | 12 | Describe how outcomes used to capture benefit(s) and harm(s) were measured. | Methods, Paragraph 4 |
| **Valuation of outcomes** | 13 | Describe the population and methods used to measure and value outcomes. | Not reported (referenced clinical paper) |
| **Measurement and valuation of resources and costs** | 14 | Describe how costs were valued. | Methods, Paragraph 3 |
| **Currency, price date, and conversion** | 15 | Report the dates of the estimated resource quantities and unit costs, plus the currency and year of conversion. | Methods, Paragraph 3 |
| **Rationale and description of model** | 16 | If modelling is used, describe in detail and why used. Report if the model is publicly available and where it can be accessed. | Not applicable |
| **Analytics and assumptions** | 17 | Describe any methods for analysing or statistically transforming data, any extrapolation methods, and approaches for validating any model used. | Not applicable |
| **Characterising heterogeneity** | 18 | Describe any methods used for estimating how the results of the study vary for subgroups. | Methods, Paragraphs 7 |
| **Characterising distributional effects** | 19 | Describe how impacts are distributed across different individuals or adjustments made to reflect priority populations. | Not considered |
| **Characterising uncertainty** | 20 | Describe methods to characterise any sources of uncertainty in the analysis. | Methods, Paragraphs 5 & 7 |
| **Approach to engagement with patients and others affected by the study** | 21 | Describe any approaches to engage patients or service recipients, the general public, communities, or stakeholders (such as clinicians or payers) in the design of the study. | Not reported |
| **Results** |  |  |  |
| **Study parameters** | 22 | Report all analytic inputs (such as values, ranges, references) including uncertainty or distributional assumptions. | Results (Table 1), Appendices 2 & 3 |
| **Summary of main results** | 23 | Report the mean values for the main categories of costs and outcomes of interest and summarise them in the most appropriate overall measure. | Results, Paragraphs 4 & 5 |
| **Effect of uncertainty** | 24 | Describe how uncertainty about analytic judgments, inputs, or projections affect findings. Report the effect of choice of discount rate and time horizon, if applicable. | Results, Paragraph 5 |
| **Effect of engagement with patients and others affected by the study** | 25 | Report on any difference patient/service recipient, general public, community, or stakeholder involvement made to the approach or findings of the study | Not reported |
| **Discussion** |  |  |  |
| **Study findings, limitations, generalisability, and current knowledge** | 26 | Report key findings, limitations, ethical or equity considerations not captured, and how these could affect patients, policy, or practice. | Discussion |
| **Other relevant information** |  |  |  |
| **Source of funding** | 27 | Describe how the study was funded and any role of the funder in the identification, design, conduct, and reporting of the analysis | End of manuscript |
| **Conflicts of interest** | 28 | Report authors conflicts of interest according to journal or International Committee of Medical Journal Editors requirements. | End of manuscript |

*From:* Husereau D, Drummond M, Augustovski F, et al. Consolidated Health Economic Evaluation Reporting Standards 2022 (CHEERS 2022) Explanation and Elaboration: A Report of the ISPOR CHEERS II Good Practices Task Force. Value Health 2022;25. https://don-husereau.shinyapps.io/CHEERS/

**Appendix 2: Unit cost resources table for costing**

| **Item** | **Unit cost (AUD$ 2020)** | **Source** |
| --- | --- | --- |
| **HOSPITALISATION** | (per day) |  |
| Inpatient stay related to febrile neutropenia | 2931.44 | [1] |
| ICU admission | 210 | [2] |
| **ANTIMICROBIALS** | Varies depending on the medication | PMCC Pharmacy Department |
| **DIAGNOSTIC IMAGING** | (per scan) |  |
| FDG PET | 953 | [MBS item number 61523](http://www9.health.gov.au/mbs/fullDisplay.cfm?type=item&q=61523&qt=item&criteria=pet) |
| X-ray chest | 47.15 | MBS item number 58503 |
| X-ray foot | 43.4 | [MBS item number 57521](http://www9.health.gov.au/mbs/fullDisplay.cfm?type=item&q=57521&qt=item&criteria=) |
| X-ray abdomen | 35.7 | [MBS item number 58900](http://www9.health.gov.au/mbs/fullDisplay.cfm?type=item&q=58900&qt=item&criteria=58900) |
| CTPA | 473.84 | MBS item number 57357 |
| HRCT | 400 | MBS item number 56307 |
| CT abdomen + pelvis (+/- neck) | 560 | MBS item number 56807 |
| CT head | 250 | MBS item number 56013 |
| CT kidneys, ureter, bladder | 155.2 | [MBS item number 57201](http://www9.health.gov.au/mbs/fullDisplay.cfm?type=item&q=57201&qt=item&criteria=) |
| CT upper abdomen | 360 | [MBS item number 56407](http://www9.health.gov.au/mbs/fullDisplay.cfm?type=item&q=56407&qt=item&criteria=) |
| CT upper abdomen + pelvis | 480.05 | [MBS item number 56507](http://www9.health.gov.au/mbs/fullDisplay.cfm?type=item&q=56407&qt=item&criteria=) |
| CT brain, chest (+/- upper abdomen) | 567.75 | [MBS item number 57007](http://www9.health.gov.au/mbs/fullDisplay.cfm?type=item&q=57007&qt=item&criteria=computed%20tomography%20chest) |
| CT pelvis | 360 | [MBS item number 56412](http://www9.health.gov.au/mbs/fullDisplay.cfm?type=item&q=56412&qt=item&criteria=tomography%20pelvis) |
| CT chest | 400 | [MBS item number 56307](http://www9.health.gov.au/mbs/fullDisplay.cfm?type=item&q=56307&qt=item&criteria=tomography%20chest) |
| CT para sinuses | 336.8 | [MBS item number 56028](http://www9.health.gov.au/mbs/fullDisplay.cfm?type=item&q=56028&qt=item&criteria=) |
| CT neck | 340 | [MBS item number 56107](http://www9.health.gov.au/mbs/fullDisplay.cfm?type=item&q=56107&qt=item&criteria=) |
| CT thoracic spine | 351.4 | [MBS item number 56225](http://www9.health.gov.au/mbs/fullDisplay.cfm?type=item&q=56225&qt=item&criteria=) |
| CT angiography | 1307.71 | [MBS item number 57353, 35321](http://www9.health.gov.au/mbs/fullDisplay.cfm?type=item&q=57353&qt=item&criteria=) |
| MRI brain | 403.2 | [MBS item number 63001](http://www9.health.gov.au/mbs/fullDisplay.cfm?type=item&q=63001&qt=item&criteria=) |
| MRI brain and cervical spine | 492.8 | [MBS item number 63111](http://www9.health.gov.au/mbs/fullDisplay.cfm?type=item&q=63111&qt=item&criteria=) |
| MRI cholangiogram | 457.2 | MBS item number 63740 |
| MRI full spine, brain | 448 | [MBS item number 63201](http://www9.health.gov.au/mbs/fullDisplay.cfm?type=item&q=63201&qt=item&criteria=) |
| MRI lumbar spine | 358.4 | [MBS item number 63151](http://www9.health.gov.au/mbs/fullDisplay.cfm?type=item&q=63151&qt=item&criteria=) |
| MRI perineum | 403.2 | [MBS item number 63743](http://www9.health.gov.au/mbs/fullDisplay.cfm?type=item&q=63743&qt=item&criteria=63743) |
| MRI thoracic and lumbar spine | 358.4 | [MBS item number 63151](http://www9.health.gov.au/mbs/fullDisplay.cfm?type=item&q=63151&qt=item&criteria=) |
| Gastrograffin follow through | 110.25 | [MBS item number 58912](http://www9.health.gov.au/mbs/fullDisplay.cfm?type=item&q=58912&qt=item&criteria=58912) |
| Nuclear medicine imaging GI bleed | 496.95 | [MBS item number 61364](http://www9.health.gov.au/mbs/fullDisplay.cfm?type=item&q=61364&qt=item&criteria=61364) |
| Nuclear medicine imaging renal | 332.5 | MBS item number 61386 |
| Nasendoscopy | 124.8 | [MBS item number 41764](http://www9.health.gov.au/mbs/fullDisplay.cfm?type=item&q=41764&qt=item&criteria=Nasendoscopy) |
| Orthopantomography | 47.4 | [MBS item number 57960](http://www9.health.gov.au/mbs/fullDisplay.cfm?type=item&q=57960&qt=item&criteria=) |
| US doppler leg vein (both) | 283.81 | [MBS item number 55886, 55244](http://www9.health.gov.au/mbs/fullDisplay.cfm?type=item&q=55886&qt=item&criteria=ultrasound%20leg) |
| US injection into joint | 152.85 | [MBS item number 55850](http://www9.health.gov.au/mbs/fullDisplay.cfm?type=item&q=55850&qt=item&criteria=55850) |
| US left upper limb | 169.5 | [MBS item number 55252](http://www9.health.gov.au/mbs/fullDisplay.cfm?type=item&q=55252&qt=item&criteria=55252) |
| US liver | 111.3 | [MBS item number 55036](http://www9.health.gov.au/mbs/fullDisplay.cfm?type=item&q=55036&qt=item&criteria=) |
| US neck | 109.1 | [MBS item number 55032](http://www9.health.gov.au/mbs/fullDisplay.cfm?type=item&q=55032&qt=item&criteria=) |
| US neck and right upper limb | 212.11 | [MBS item number 55032, 55864](http://www9.health.gov.au/mbs/fullDisplay.cfm?type=item&q=55032&qt=item&criteria=) |
| US pelvis female | 98.25 | [MBS item number 55065](http://www9.health.gov.au/mbs/fullDisplay.cfm?type=item&q=55065&qt=item&criteria=) |
| US renal tract | 109.1 | [MBS item number 55038](http://www9.health.gov.au/mbs/fullDisplay.cfm?type=item&q=55038&qt=item&criteria=) |
| US right chest wall | 109.1 | [MBS item number 55812](http://www9.health.gov.au/mbs/fullDisplay.cfm?type=item&q=55812&qt=item&criteria=) |
| US right foot | 109.1 | [MBS item number 55892](http://www9.health.gov.au/mbs/fullDisplay.cfm?type=item&q=55892&qt=item&criteria=) |
| US right upper limb | 103.01 | [MBS item number 55864](http://www9.health.gov.au/mbs/fullDisplay.cfm?type=item&q=55864&qt=item&criteria=) |
| US salivary gland | 109.1 | [MBS item number 55030](http://www9.health.gov.au/mbs/fullDisplay.cfm?type=item&q=55030&qt=item&criteria=) |
| US shoulder | 103.01 | [MBS item number 55864](http://www9.health.gov.au/mbs/fullDisplay.cfm?type=item&q=55864&qt=item&criteria=) |
| US testes | 109.5 | [MBS item number 55048](http://www9.health.gov.au/mbs/fullDisplay.cfm?type=item&q=55048&qt=item&criteria=) |
| US upper abdomen (+/-) urinary tract | 111.3 | [MBS item number 55036](http://www9.health.gov.au/mbs/fullDisplay.cfm?type=item&q=55036&qt=item&criteria=) |
| Ventilation perfusion scan | 443.35 | [MBS item number 61348](http://www9.health.gov.au/mbs/fullDisplay.cfm?type=item&q=61348&qt=item&criteria=ventilation%20perfusion) |
| **INVASIVE DIAGNOSTICS** | (per procedure) |  |
| Diagnostic biopsy |  |  |
| Skin | 53.05 | MBS item number 30071 |
| Lymph node | 152.15 | MBS item number 30075 |
| Punch biopsy of vulva lesion | 66.45 | MBS item numbers 36515 |
| Bronchoscopy including anaesthesia | 381.9 | MBS item numbers 41889, 20520, 23045 |
| Echocardiography |  |  |
| TTE (Transthoracic) | 214.33 | MBS item number 55126 |
| Surgical procedures |  |  |
| Bilateral JJ stent insertion | 974.45 | MBS item numbers 36633, 20806, 20806 |
| CT guided lung biopsy | 373.3 | MBS item numbers 38812, 20522, 20806 |
| L. intercostal catheter insertion | 414.55 | MBS item numbers 38436, 20522, 20806 |
| Angioembolisation of SMA | 1007.2 | MBS item numbers 35410, 20740, 20806 |
| Colonoscopy | 339.7 | MBS item numbers 32223 |
| Colonoscopy with APC | 1623.7 | MBS item numbers 30684, 20810, 20806 |
| Lung wedge resection | 1587.65 | MBS item numbers 38440, 20540, 23085 |
| Sigmoidoscopy | 213.65 | MBS item numbers 32084, 20900, 23025 |
| Subdural haematoma evac | 602.1 | MBS item numbers 31551, 20305, 20806 |
| Tooth extraction | 55.8 | MBS item numbers 75200 |
| **MICROBIOLOGY PATHOLOGY TESTS** | (per test) |  |
| Blood culture | 30.75 | MBS item number 69354 |
| Laboratory pathology tests |  |  |
| Stool | 52.9 | MBS item number 69345 |
| Blood | 30.75 | MBS item number 69354 |
| Hickman site | 48.15 | MBS item number 69321 |
| Mouth | 22 | MBS item number 69303 |
| Other | 48.15 | MBS item number 69321 |
| Pleural | 48.15 | MBS item number 69321 |
| Skin | 33.75 | MBS item number 69306 |
| Sputum | 33.75 | MBS item number 69318 |
| Urine | 43 | MBS item number 69324 |
| Urogenital | 33.75 | MBS item number 69312 |
| Vulval biopsy | 33.75 | MBS item number 69312 |
| Cytology | 48.6 | MBS item number 73045 |
| PCR | 28.65 | MBS item number 69494 |
| Fungal culture | 48.15 | MBS item number 69309 |
| Panfungal PCR | 133.58 | [2] |
| **CVC RE-INSERTION** | (per procedure) |  |
| Hickman or Permacath insertion | 560.45 | MBS item number 34527 |
| PICC insertion | 115.45 | MBS item number 13815 |

[1] Lingaratnam S, Thursky K, Slavin M, Kirsa S, Bennett C, Worth L. The disease and economic burden of neutropenic fever in adult patients in Australian cancer treatment centres 2008: analysis of the Victorian Admitted Episodes Dataset. Internal medicine journal. 2011;41(1b):121-9.

[2] Independent Health and Aged Care Pricing Authority. National pricing model: technical specifications 2019–20: Independent Hospital Pricing Authority; 2019 [accessed 15 March 2022]. Available from: <https://www.ihacpa.gov.au/health-care/pricing/national-pricing-model-technical-specifications>.

[3] Garnham K, Halliday CL, Kok J, Jayawardena M, Ahuja V, Green W, et al. Knowledge at what cost? An audit of the utility of panfungal PCR performed on bronchoalveolar lavage fluid specimens at a tertiary mycology laboratory. Pathology. 2020;52(5):584-8.

**Appendix 3: Resources used across both cohorts**

|  | **FDG-PET-CT group (n=65)** | | | | | **Standard CT group (n=69)** | | | | |
| --- | --- | --- | --- | --- | --- | --- | --- | --- | --- | --- |
|  | **Mean** | **Min** | **Max** | **Median** | **IQR** | **Mean** | **Min** | **Max** | **Median** | **IQR** |
| **Antimicrobials ^a^** | 5.3 | 1 | 23 | 5 | 2 | 4.4 | 1 | 10 | 4 | 2 |
| **Diagnostic imaging ^b^** | 0.9 | 0 | 4 | 0 | 2 | 1.3 | 0 | 5 | 1 | 2 |
| **Invasive diagnostics ^c^** | 0.2 | 0 | 3 | 0 | 0 | 0.3 | 0 | 3 | 0 | 0 |
| **Pathology tests** | 6.9 | 0 | 50 | 5 | 9 | 9.9 | 0 | 71 | 6 | 11 |
| **CVC reinsertion** | 0.2 | 0 | 2 | 0 | 0 | 0.2 | 0 | 3 | 0 | 0 |
| **ICU (days)** | 2.0 | 0 | 60 | 0 | 0 | 1.0 | 0 | 24 | 0 | 0 |
| **Total duration of hospitalisation (days)** | 15.4 | 1 | 67 | 10 | 10 | 17.3 | 3 | 80 | 13 | 10 |

^a^ Related to treatment for febrile neutropenia episode

^b^ Includes x-rays, CT, MRI scans, echocardiograms. Does not include intervention FDG-PET-CT scan

^c^ Includes biopsies, bronchoscopies and any relevant surgical procedures

**Appendix 4: Cost-effectiveness analysis results**

**Figure SM4.1: Cost-effectiveness analysis plane for 6-month mortality outcome**

The red dot represents the ICER point estimate which sits in the south-west quadrant, indicating that the FDG-PET-CT intervention is cheaper and results in less deaths (more deaths averted) compared to standard CT.


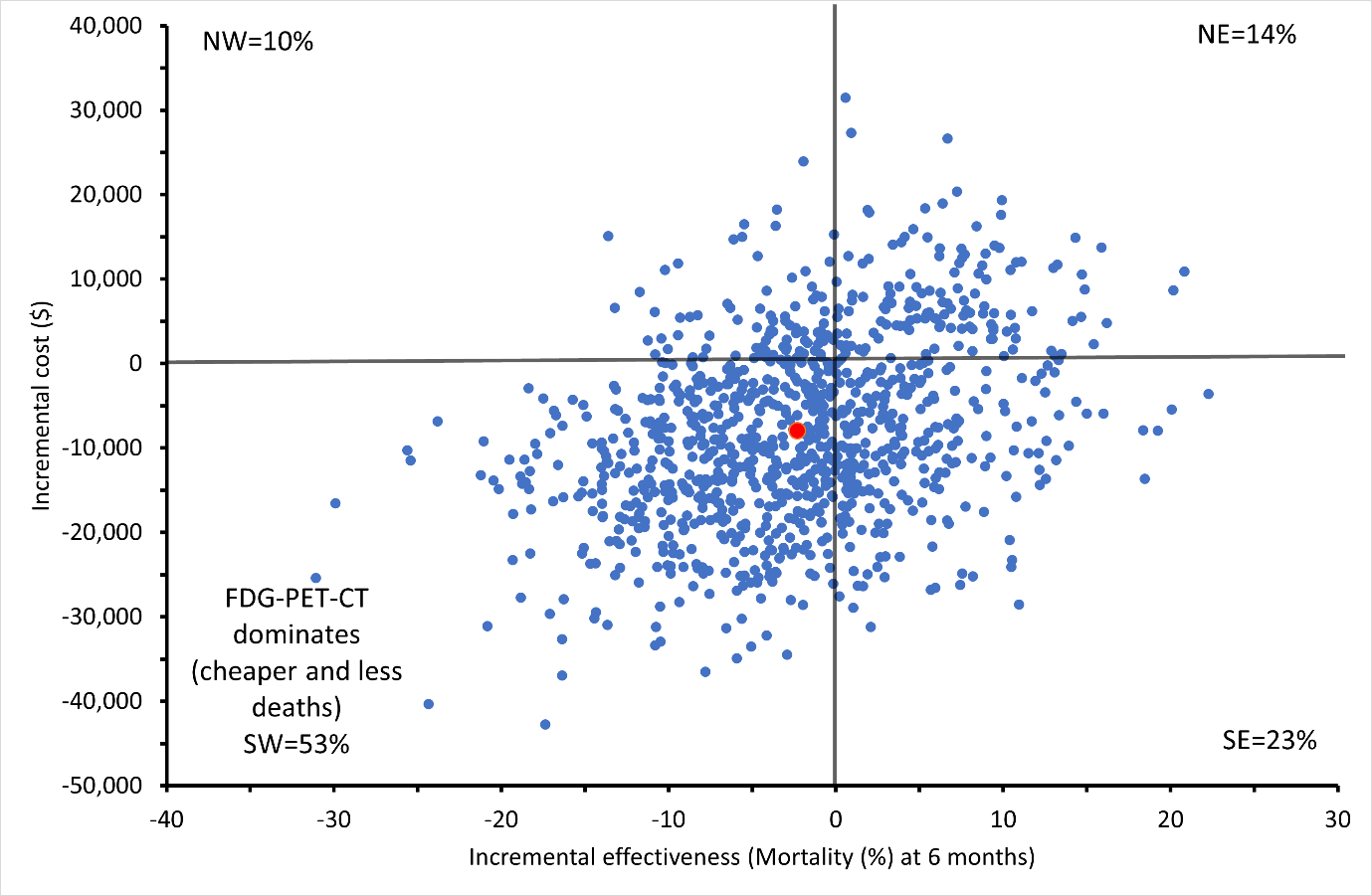


NW= north-west quadrant; intervention more costly, less deaths observed

NE= north-east quadrant; intervention more costly, more deaths observed

SE= south-east quadrant; intervention less costly, more deaths observed

SW= south-west quadrant; intervention less costly, less deaths observed

**Appendix 5: Subgroup analysis results**

Post-hoc subgroup analysis by reason of admission (chemotherapy or transplant)

|  | **FDG-PET-CT** | **Standard CT** | **Difference** | **ICER ($/QALY)** |
| --- | --- | --- | --- | --- |
| **Chemotherapy** | | | | |
| Total cost ($) | 45,540 | 52,963 | -7,423 | FDG-PET-CT dominates ^a^ |
| Outcomes (QALY) | 0.285 | 0.284 | 0.002 |  |
| **Transplant** | | | | |
| Total cost ($) | 49,396 | 57,448 | -8,052 | FDG-PET-CT dominates ^a^ |
| Outcomes (QALY) | 0.286 | 0.284 | 0.002 |  |

^a^ FDG-PET-CT dominates indicates that FDG-PET-CT is less costly and more effective

**Appendix 6: Sensitivity analyses results**

To explore parameter uncertainty, several sensitivity analyses were conducted, and results (cost per QALY gained) presented in table below and on cost-effectiveness plane. The variables tested include:

- SA1: Excluding patient with prolonged ICU stay
- SA2: Increasing cost of FDG-PET-CT (intervention) by 20%
- SA3: Increase cost of FDG-PET-CT (intervention) by 100%
- SA4: Increase cost of hospitalisation by 20%
- SA5: Decrease cost of hospitalisation by 20%
- SA6: Decrease in-hospital utility value by 0.5 SD
- SA7: Decrease out-of-hospital utility value by 0.5 SD
- SA8: Decrease both in-hospital and out-of-hospital utility values by 0.5 SD

**Table SM6.1: Sensitivity analyses results**

|  | **FDG-PET-CT** | **Standard CT** | **Difference** | **ICER ($/QALY)** |
| --- | --- | --- | --- | --- |
| **SA1: Excluding patient with prolonged ICU stay** | | | | |
| Total cost ($) | 43,971 | 59,048 | -15,077 | FDG-PET-CT dominates ^a^ |
| Outcomes (QALY) | 0.286 | 0.284 | 0.002 |  |
| **SA2: Increasing cost of FDG-PET-CT (intervention) by 20%** | | | | |
| Total cost ($) | 49,638 | 57,300 | -7,662 | FDG-PET-CT dominates ^a^ |
| Outcomes (QALY) | 0.285 | 0.284 | 0.001 |  |
| **SA3: Increase cost of FDG-PET-CT (intervention) by 100%** | | | | |
| Total cost ($) | 50,668 | 57,467 | -6,799 | FDG-PET-CT dominates ^a^ |
| Outcomes (QALY) | 0.285 | 0.284 | 0.001 |  |
| **SA4: Increase cost of hospitalisation by 20%** | | | | |
| Total cost ($) | 56,465 | 67,789 | -11,324 | FDG-PET-CT dominates ^a^ |
| Outcomes (QALY) | 0.285 | 0.284 | 0.001 |  |
| **SA5: Decrease cost of hospitalisation by 20%** | | | | |
| Total cost ($) | 41,610 | 47,613 | -6,003 | FDG-PET-CT dominates ^a^ |
| Outcomes (QALY) | 0.285 | 0.284 | 0.001 |  |
| **SA6: Decrease in-hospital utility value by 0.5 SD** | | | | |
| Total cost ($) | 49,174 | 57,624 | -8,450 | FDG-PET-CT dominates ^a^ |
| Outcomes (QALY) | 0.281 | 0.279 | 0.002 |  |
| **SA7: Decrease out-of-hospital utility value by 0.5 SD** | | | | |
| Total cost ($) | 49,174 | 57,624 | -8,450 | FDG-PET-CT dominates ^a^ |
| Outcomes (QALY) | 0.234 | 0.234 | 0.001 |  |
| **SA8: Decrease both in-hospital and out-of-hospital utility values by 0.5 SD** | | | | |
| Total cost ($) | 49,174 | 57,624 | -8,450 | FDG-PET-CT dominates ^a^ |
| Outcomes (QALY) | 0.230 | 0.229 | 0.001 |  |

^a^ FDG-PET-CT dominates indicates that FDG-PET-CT is less costly and more effective

**Figure SM6.1: Cost-effectiveness plane showing results of sensitivity analyses (SA1-SA4)**


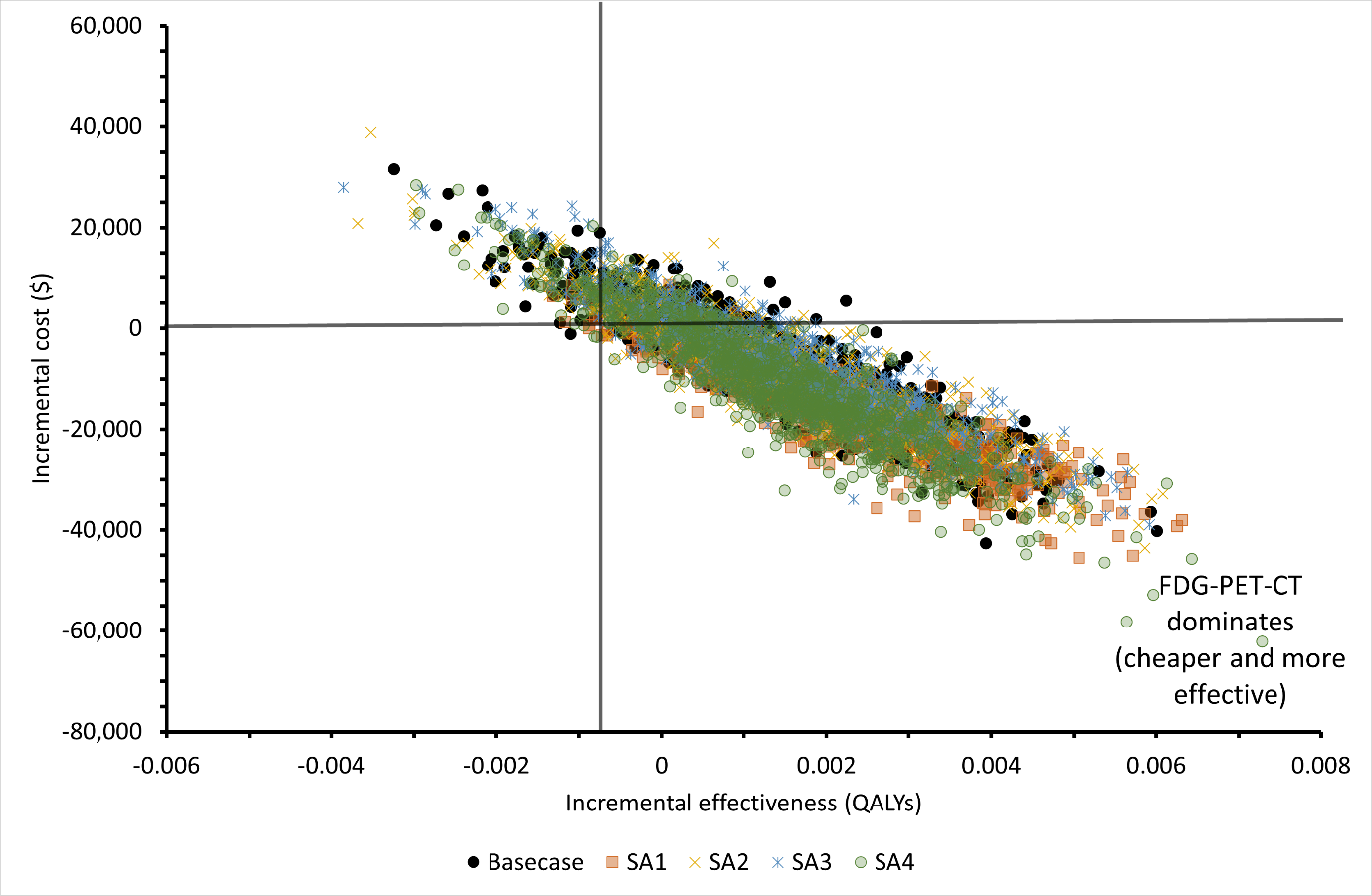


**Figure SM6.2: Cost-effectiveness plane showing results of sensitivity analyses (SA5-SA8)**


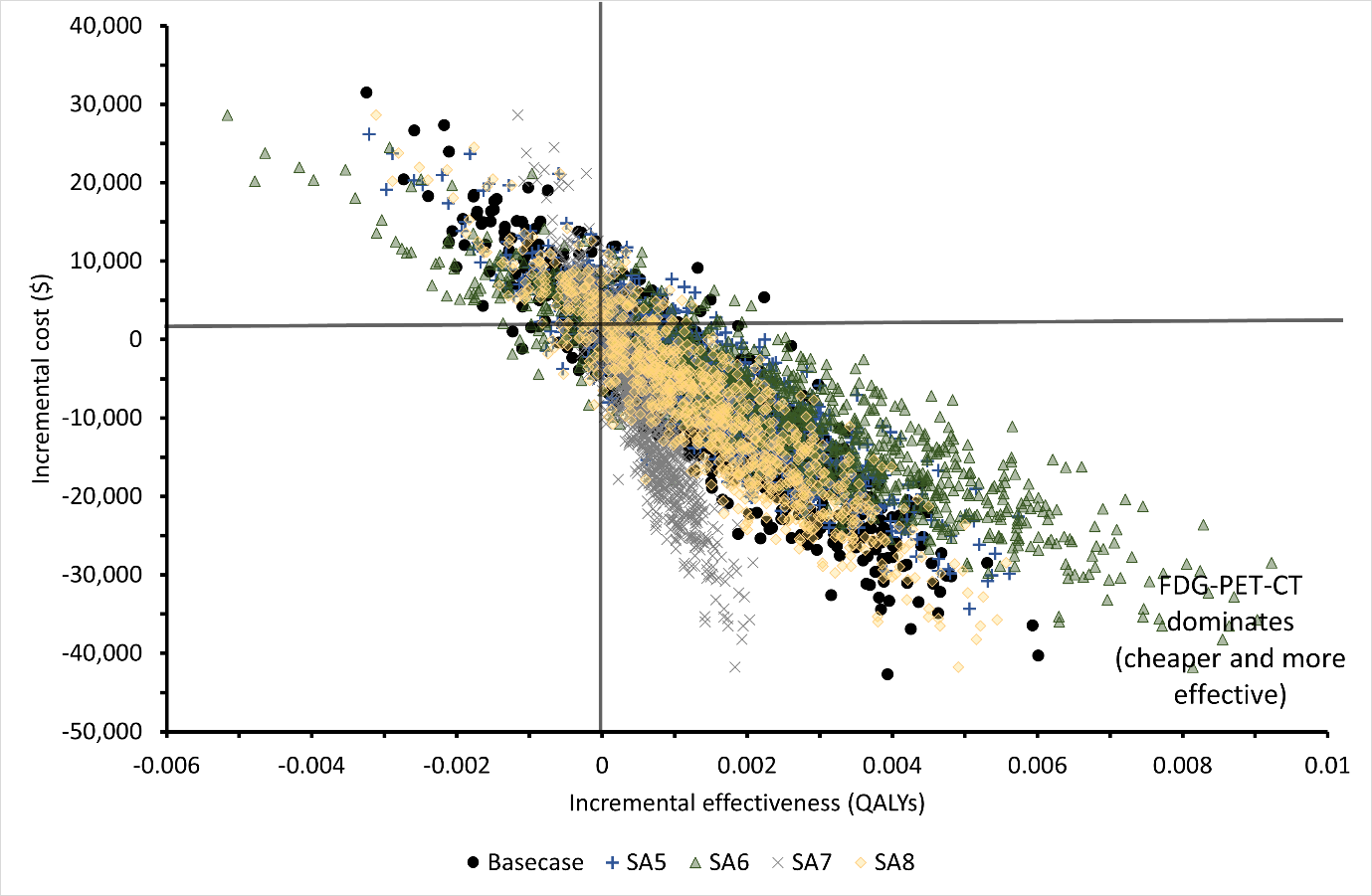

Supplement: Supplementary file 1 — Supplementary Material 1: Appendix 1 – 6 [file 40644_2023_647_MOESM1_ESM.docx]
